# Caspofungin-Non-Susceptible *Candida orthopsilosis* Isolated from Onychomycosis in Iran

**Published:** 2017-02

**Authors:** Rasoul MOHAMMADI, Hossein MIRHENDI, Mohammad Taghi HEDAYATI, Hamid BADALI

**Affiliations:** 1. Dept. of Medical Parasitology and Mycology, School of Medicine, Isfahan University of Medical Sciences, Isfahan, Iran; 2. Infectious Diseases and Tropical Medicine Research Center, Isfahan University of Medical Sciences, Isfahan, Iran; 3. Dept. of Medical Parasitology and Mycology, School of Public Health, National Institute of Health Research, Tehran University of Medical Sciences, Tehran, Iran; 4. Dept. of Medical Mycology and Parasitology, School of Medicine, Mazandaran University of Medical Sciences, Sari, Iran; 5. Invasive Fungi Research Center (IFRC), School of Medicine, Mazandaran University of Medical Sciences, Sari, Iran

**Keywords:** *Candida orthopsilosis*, Onychomycosis, Antifungal susceptibility, Iran

## Abstract

**Background:**

Although *Candida albicans* remains the most common fungal isolate from clinical specimens, many studies have detected a shift towards non-albicans *Candida* species. Despite worrying clinical pictures associated with latter species*,* there is little information regarding its susceptibility patterns against currently available antifungal agents, with only a small number of strains having been studied.

**Methods:**

We evaluated the in vitro antifungal susceptibilities of clinical isolates of *C. orthopsilosis* already identified by two-steps PCR-RFLP and reconfirmed by sequence analysis of entire ITS rDNA region, to six antifungal drugs.

**Results:**

The resulting MIC_50_ and MIC_90_ for all strains (n=18) were in increasing order, as follows: posaconazole (0.016 & 0.063 μg/ml); itraconazole (0.031 & 0.125 μg/ml); amphotericin B (0.5 & 1 μg/ml); fluconazole (0.25 & 0.5 μg/ml) and caspofungin (4 & 8 μg/ml). A uniform pattern of the MIC ranges was seen for amphotericin B, fluconazole, itraconazole, and posaconazole, while a widest range and the highest MICs were observed for caspofungin.

**Conclusion:**

Although we emphasis on the careful species designation of the clinical isolates of *Candida*, the antifungal susceptibility patterns of these clinically important organisms may have an application in clinical and epidemiological setting and deserve the implementation of local surveillance programs to monitor.

## Introduction

*Candida albicans* has been considered the main cause of *Candida* infections, but non-*albicans Candida* species, such as *C. glabrata, C. tropicalis, C. parapsilosis* complex, and *C. krusei* are also significant pathogens (1, 2). Considering the variable antifungal susceptibility profiles of different *Candida* species and the emergence of diseases due to rare *Candida* species, correct species delineation is necessary for clinicians to make a well-documented therapeutic decision (3–6). *Candida parapsilosis* was a complex of three genetic groups designated as I, II and III. This classification was based on several molecular studies and according to the results from various approaches. Subsequently according to a multilocus sequence typing scheme, *C. parapsilosis* groups I, II and III should be reclassified as the species *C. parapsilosis, C. orthopsilosis* and *C. metapsilosis*, respectively (4). Among the most common *Candida* species involved in invasive candidiasis, *C. parapsilosis sensu lato* is particularly common in neonates, in catheter-associated candidaemia and in association with intravenous hyper alimentation, as well as in superficial candidiasis such as onychomycosis (7, 8).

A small proportion of some isolates are actually the closely related species *C. orthopsilosis* or *C. metapsilosis*, with a significant geographical variation (4, 9). Although, *C. parapsilosis* group is normally susceptible to most antifungal compounds, elevated MICs for the echinocandin agents are consistently reported for this species, probably due to an intrinsic mutation in the FKS hot spot (10). An echinocandin MIC of >8 μg/ml is used to identify caspofungin-resistant *C. parapsilosis* and susceptibility differences within the *C. parapsilosis* group could affect therapeutic choices (11). Unfortunately, CLSI clinical breakpoint (CBP) values for *C. orthopsilosis* is unclear. Recently we reported the species distribution of several hundred clinical isolates of yeasts, obtained from four different provinces in Iran. A set of polymerase chain reaction (PCR) amplification of ITS1-5.8S-ITS2 region of rDNA and secondary alcohol dehydrogenase (SADH) gene, followed by restriction fragment length polymorphism (RFLP) for each targets was used for species delineation of the isolates. In this study, *C. parapsilosis* complex were the second most common (after *C. albicans*) isolates and the most abundant of non-*albicans Candida* isolates. Overall, 128 strains of *C. parapsilosis* group including 110 *C. parapsilosis sensu stricto* and 18 *C. orthopsilosis* were isolated from different kinds of clinical samples mostly from nails scrapings.

In the present study we focused on the *C. orthopsilosis* strains. The identity of these isolates was reconfirmed by ITS rDNA-sequencing and their *in vitro* antifungal susceptibility profile was conducted for several antifungal drugs.

## Materials and Methods

All *C. orthopsilosis* strains used in this study were screened from several hundred clinical yeast isolates, which already delineated to the species level among 2009 to 2011 from patients with various clinical forms of candidiasis (8). *C. orthopsilosis* were screened as described previously (12). Furthermore to confirm the species recognized as *C. orthopsilosis*, a total of five ITS-PCR products relevant to five *C. orthopsilosis* strains, were randomly selected and subjected to identifying by sequencing the ITS1-5.8S-ITS2 region with the universal primers ITS1 and ITS4 (13). Sequencing was performed by using an ABI 3730XL automatic sequencer (Applied Biosystems, Foster City, CA, U.S.A.). The consensus edited sequences were subjected to nucleotide BLAST search (http://blast.ncbi.nlm.nih.gov/Blast.cgi) and DNASIS multiple alignment analysis.

### In vitro antifungal susceptibility testing

Minimum inhibitory concentration (MICs) was determined according to recommendations stated in the clinical and laboratory standards institute (CLSI) M27-A3 and M27-S4 documents (14, 15). Amphotericin B (Bristol-Myers-Squib, Woerden, and the Netherlands), fluconazole (Pfizer Central Research, Sandwich, United Kingdom), itraconazole (Janssen Research Foundation, Beerse, Belgium), posaconazole (Schering-Plough, Kenilworth, USA) and caspofungin (Merck Sharp & Dohme, Haarlem, The Netherlands) were used for preparation of the CLSI microdilution trays. The antifungal agents were diluted in the standard RPMI-1640 medium (Sigma Chemical Co.) buffered to pH 7.0 with 0.165 M morpholinepropanesulfonic acid (MOPS) (Sigma) with L-glutamine without bicarbonate. To yield two times concentrations and dispensed into 96-well microdilution trays at a final concentration of 0.016–16 μg/ml for amphotericin B, itraconazole, posaconazole; 0.063–64 μg/ml for fluconazole and 0.08–8 μg/ml for caspofungin. Plates were stored at −70 °C until use. All identified *C. orthopsilosis* were grown on malt extract agar (MEA, Difco) plates at 35 °C and inoculum suspensions were prepared by harvesting the cell from 24 h old cultures and were adjusted spectrophotometrically in saline to optical densities ranged 75–77% transmission. Final inoculum sizes ranged from 2.5×10^3^ to 5×10^3^ CFU/ml as demonstrated by a quantitative colony count on Sabouraud’s dextrose agar. MIC results for all agents were read following 24 h of incubation at 35 °C. MIC values were determined visually, as the lowest concentration of drug that caused complete (amphotericin B) or significant (>50%) growth diminution levels (all other agents) (14). *C. parapsilosis* ATCC 22019 and *C. krusei* ATCC 6258 were chosen as quality controls to be used with every new series of MICs plates. Based on newly revised CLSI clinical breakpoint (CBP) for caspofungin MIC values of <0.12 and >0.5 μg/ml were considered susceptible and resistant, respectively. For *C. glabrata*; fluconazole MIC results of <2 and >8 μg/ml were defined as susceptible and resistant, respectively. For *C. albicans*, *C. parapsilosis*, and *C. tropicalis*, and MICs of <32 and >64 μg/ml were considered susceptible dose dependent (SDD) and resistant, respectively. For *C. glabrata* susceptible and resistant breakpoints for voriconazole are <0.12 and >1 μg/ml, respectively. For *C. albicans*, *C. parapsilosis*, and *C. tropicalis*, CLSI has not assigned CBPs for voriconazole and *C. glabrata* and recommends the ECV of 0.5 μg/ml to be used to differentiate wild type (WT; MIC < ECV) from non-WT (MIC>ECV) strains of this species (15, 16).

### Statistical Analysis

The MIC range, geometric, MIC_50_ and MIC_90_ were performed. The MIC_50_ and MIC_90_ values were calculated as the minimum concentrations of agents that were able to inhibit 50% and 90% of the isolates, respectively. The high and low off-scale MICs were included in the analysis by conversion to the next higher and lower antifungal concentrations, based on CLSI breakpoint (14–16). Data were analyzed using the SPSS software ver. 14.0 (Chicago, IL, USA). Comparison of species distribution and antifungal susceptibility rates was adjusted using Fisher’s exact test and Mann-Whitney *U-*test. A *P*-value of < 0.05 was considered significant.

## Results

**Fig. 1:**
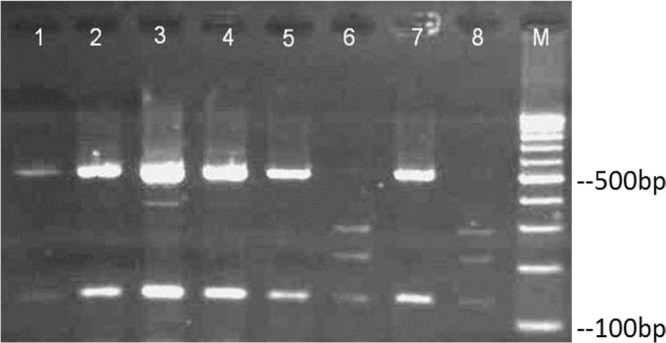
Digestion of SADH-PCR products with *Nla*III restriction enzyme in the group of *C. parapsilosis* Lane 1, 2, 3, 4, 5, 7: *C. parapsilosis*, and lanes 6 and 8: *C. orthopsilosis*

Among 128 isolates genetically identified as *C. parapsilosis sensu stricto,* 18 strains were recognized to be *C. orthopsilosis* (Table 1). The remaining was *C. parapsilosis sensu stricto* but no *C. metapsilosis* was found. Five isolates were randomly selected for ITS rDNA sequence analysis. The results confirmed the species that recognized by PCR-RFLP (Fig. 1).

**Table 1:** Species distribution of *Candida parapsilosis*, *C. orthopsilosis,* and *C. metapsilosis* evaluated in this study

**Clinical origin**	***C. parapsilosis***	***C. orthopsilosis***	***C. metapsilosis***
Nail samples	80 (62.5%)	15 (11.7%)	0
Blood cultures	5 (4%)	0	0
Vaginal discharges	1 (0.7%)	0	0
Skin scrapping	18 (14.1%)	2 (1.4%)	0
BAL	1 (0.7%)	0	0
Sputum	1 (0.7%)	0	0
Esophageal biopsy	1 (0.7%)	0	0
Ear discharge	3 (2.3%)	0	0
Peritoneal fluid	0	1 (0.7%)	0

The sequences were deposited in GenBank with the accession numbers JQ366004-JQ366008. Table 2 summarized the *in vitro* antifungal susceptibility testing of the *C. orthopsilosis* isolates.

**Table 2:** In vitro susceptibilities of 18 clinical isolates of *C. orthopsilosis* to five antifungal agents

**Antifungal agents**	**MIC Range**	**MIC_50_**	**MIC_90_**	**G mean**
Amphotericin B	0.25–2	0.5	1	0.3353
Fluconazole	0.25–2	0.25	0.5	0.3535
Itraconazole	0.016–0.25	0.031	0.125	0.0622
Posaconazole	0.002–0.063	0.016	0.063	0.0317
Caspofungin	1–8	4	8	3.474

MIC range, geometric (G) mean, MIC_50_ and MIC_90_ values expressed in μg/ml

A uniform pattern of the MIC ranges were seen for amphotericin B (0.25–2 μg/ml), fluconazole (0.25–2 μg/ml), itraconazole (0.016–0.25 μg/ml) and posaconazole (0.002–0.063 μg/ml), while a widest range (1–8 μg/ml) and the highest MICs (8 μg/ml) were observed for caspofungin. In terms of MIC_50_ (0.016 μg/ml), and MIC_90_ (0.063μg/ml) posaconazole was more active than both fluconazole (MIC_50_ 0.25 and MIC_90_ 0.5 μg/ml) and itraconazole (MIC_50_ 0.031 and MIC_90_ 0.125 μg/ml). Fluconazole exhibited the potent activity against *C. orthopsilosis* and fluconazole resistance (MIC > 8 μg/ml) and susceptible dose dependent (SDD) (4 μg/ml) were not observed among the isolates. In terms of MIC_50_ and MIC_90_ caspofungin (4 and 8 μg/ml) was higher than other antifungal agents and all isolates were highly resistant to caspofungin.

## Discussion

Molecular screening of 128 *C. parapsilosis* strains isolated from clinical sources (mainly from nail) in Iran revealed that 14% of infections are ascribed to *C. orthopsilosis.* In accordance with the present study, *C. metapsilosis* has been infrequently recovered from clinical samples (17, 18), while *C. orthopsilosis* was recovered from blood, nails, skin, lungs, urine, and catheters (19). The higher prevalence of *C. parapsilosis* may be related to its ubiquitous nature, since it is commonly isolated from different environmental sources or might be related to larger virulence factors compared to two other relative species (12). However, this still needs to be entirely confirmed. Interestingly, prevalence of *C. orthopsilosis* is very high in comparison with the same studies in other countries. For instance, only one *C. orthopsilosis* isolate without any *C. metapsilosis* was reported (20). Any *C. orthopsilosis* found among the isolates (21). Thirteen out of 240 isolates (4.5%) were revealed of *C. orthopsilosis* isolates without any *C. metapsilosis* (19), any *C. orthopsilosis* found among 395 isolates but reported 20 (5.1%) strains of *C. metapsilosis* (22). Higher frequency of *C. orthopsilosis* was reported, 28.1% *C. orthopsilosis* isolates (4) and 24.4% in comparison with our survey (14%) (23). The prevalence and distribution of the species in the *C. orthopsilosis* caused candidiasis is not yet clear. However, we found that at least a significant number of *C. orthopsilosis* could be from superficial lesions as reported in other studies (19, 24–26). DNA-based methods provide data that are more accurate and reproducible than visual characteristics and phenotypic properties. In addition, by given the expansion of non-*Candida albicans* yeast infections, and the distinct antifungal susceptibility profile of the related species, accurate and precise identification becomes essential for clinical management of the patients.

In vitro antifungal susceptibility of *C. orthopsilosis* against fluconazole showed similar susceptibility profiles in comparison to the results (16, 25). The high MICs to caspofungin of *C. orthopsilosis* isolates in our study agree to the previous findings (18, 25). However, few studies showed limited evidence of resistance to caspofungin against clinical isolates of *C. orthopsilosis* (28–30). An MIC of caspofungin of >4 μg/ml only in 6 (0.1%) of 5346 isolates tested without any evidence of increasing caspofungin resistance over the last 6 yr of their study (31). In our study, an MIC of caspofungin based on both recently revised CLSI clinical breakpoint value and old clinical breakpoint value (14) of >8 μg/ml was observed in four isolates tested. In terms of MIC_50_ and MIC_90_ patterns obtained in the current study suggested that *C. orthopsilosis* is resistant to caspofungin. Although limited by small numbers, this study provides additional information of resistant *C. orthopsilosis* obtained from onychomycosis. However, their clinical effectiveness in the treatment of *C. orthopsilosis* infections remains to be determined.

## Conclusion

We emphasis on the careful species designation of the clinical isolates of *Candida,* specially *Candida metapsilosis* and *C. orthopsilosis* as cryptic species from *C. parapsilosis* with different prevalence rates, virulence and in vitro susceptibility profiles. Although clinical resistance to azoles has been increasingly reported in *C. parapsilosis,* in the current study, all tested *C. orthopsilosis* isolates were found to be susceptible to fluconazole and non-susceptible to caspofungin. It seems that with increasing usage of echinocandins, the epidemiology and resistance distribution of *C. orthopsilosis* deserve attention.

## Ethical considerations

Ethical issues (Including plagiarism, informed consent, misconduct, data fabrication and/or falsification, double publication and/or submission, redundancy, etc.) have been completely observed by the authors.
